# The SGLT2 Inhibitor Empagliflozin Mitigates the Harmful Effects of Methylglyoxal Exposure on Ovalbumin-Induced Mouse Airway Inflammation

**DOI:** 10.3390/ijms26125753

**Published:** 2025-06-16

**Authors:** Matheus L. Medeiros, Akila L. Oliveira, Edson Antunes

**Affiliations:** Department of Pharmacology, Faculty of Medical Sciences, University of Campinas (UNICAMP), Alexander Fleming St., Campinas 13083-881, SP, Brazil; a192906@dac.unicamp.br (A.L.O.); eantunes@unicamp.br (E.A.)

**Keywords:** asthma, cytokines, mucus, collagen, glyoxalase, MG-H1

## Abstract

Asthma is a chronic inflammatory airway disease that can be aggravated by metabolic comorbidities such as type 2 diabetes mellitus (DM2) and obesity. Elevated levels of methylglyoxal (MGO), a reactive glycolysis byproduct, have been associated with exacerbation of allergic airway disease. SGLT2 inhibitors have been successfully employed in DM2 treatment. Here, we hypothesized that elimination of MGO might be a potential anti-inflammatory mechanism of SGLT2 inhibitors. This study aimed to evaluate the effects of empagliflozin on ovalbumin (OVA)-induced airway inflammation in mice chronically exposed to MGO. Male C57BL/6 mice sensitized with OVA were exposed to 0.5% MGO for 12 weeks and treated with empagliflozin (10 mg/kg, gavage, two weeks). MGO exposure significantly enhanced airway eosinophil infiltration, mucus production and collagen deposition, as well as levels of IL-4, IL-5, eotaxin and TNF-α. Empagliflozin treatment significantly reduced OVA-induced airway disease, which was accompanied by reductions in IgE, IL-4, IL-5, eotaxin, and TNF-α levels. Empagliflozin significantly reduced the MGO levels in serum, and immunohistochemical staining, and protein expression of MGO-hydroimidazolone (MG-H1), while increasing IL-10 levels and glyoxylase-1 (GLO 1) activity in lungs. In conclusion, empagliflozin efficiently removes MGO from circulation, while increasing the MGO detoxification by GLO 1, thereby mitigating the OVA-induced inflammation in MGO-exposed mice.

## 1. Introduction

Asthma is a chronic inflammatory disorder of the airways, characterized by persistent airway inflammation, mucus hypersecretion, and bronchial hyperresponsiveness, leading to variable airflow limitations. Clinically, it manifests as recurrent episodes of wheezing, dyspnea, chest tightness, and coughing, which may vary in intensity and frequency over time [1]. Many cell types including dendritic cells, lymphocytes, innate lymphoid cells (ILCs), eosinophils, neutrophils, and mast cells contribute to the pathophysiology of asthma, which interact with structural cells such as epithelial cells, leading to the development of asthma. However, today, eosinophils are recognized as pivotal cells for the allergic airway disease. Eosinophils actively modulate the allergic inflammatory response through the release of cytokines and neuropeptides, which perpetuate inflammation, worsening the disease progression [2,3]. Classical allergic asthma, associated with a type 2 immunity response, is predominantly driven by the activation of T helper 2 (Th2) lymphocytes, which orchestrate a cascade of pro-inflammatory events characteristic of the type 2 immunity-high phenotype [4], as characterized by excessive production of immunoglobulin E (IgE) and type 2 cytokines such as IL-4, IL-5 and IL-13, as well as eotaxin-1, a selective eosinophil chemoattractant CCL11 chemokine, all of which play crucial roles in asthma [2,3,4,5]. A variety of additional mediators, such as TNF-α [6] and IL-17 [7], have been implicated in the pathogenesis of asthma by promoting the release of pro-inflammatory molecules and chemotactic signals that facilitate immune cell recruitment and contribute to airway remodeling. Conversely, IL-10 produced by macrophages and other cell types exerts an immunoregulatory role by suppressing type 2 cytokine production and downregulating pro-inflammatory gene expression, thereby attenuating allergic inflammation [8].

A widely used preclinical model of allergic asthma that replicates key pathophysiological features of the human condition, mainly the eosinophilic infiltration of the airways, involves immunization and repeated intranasal challenges with ovalbumin (OVA) in mice [9]. Among the various factors known to influence asthma severity, obesity, and poorly controlled type 2 diabetes mellitus have emerged as significant comorbidities that exacerbate the disease progression and diminish the responsiveness to conventional pharmacological therapies, as demonstrated in human studies [10] and animal models [11,12,13,14,15]. Considering that obesity has been recognized as a low-grade chronic inflammation that may precede asthma, it is accepted that the large airway infiltration in diabetic/obese individuals is a consequence of overproduction of type 1/type 2 cytokines, chemokines, and adipokines, but much about the relationship between obesity and asthma remains unclear [12,16].

Methylglyoxal (MGO) is one of the most reactive endogenously produced dicarbonyl compounds [17,18]. It is primarily formed as a byproduct of glycolysis, arising from the non-enzymatic degradation of glyceraldehyde-3-phosphate and dihydroxyacetone phosphate [19]. Under physiological conditions, MGO is primarily detoxified by the glyoxalase (GLO) system, which includes GLO 1 and GLO 2 enzymes [20]. MGO promotes post-translational modification of peptides or proteins, leading to generation of advanced glycation end-products (AGEs) [21], which activate a cell surface receptor termed RAGE that is coupled to multiple intracellular signaling pathways, including generation of high levels of oxidative stress [22]. Persistent elevation of MGO concentration in blood and urine has been observed in various metabolic disorders, mainly prediabetes, diabetes mellitus, and obesity [23,24], which is implicated in several pathological conditions in humans and animal models such as renal injury [25], cardiovascular diseases [26,27], retinopathy [28], bladder overactivity [29], cancer [30], neurodegenerative diseases [31], and chronic inflammatory conditions [32]. In addition, prolonged MGO consumption in healthy normoglycemic mice significantly potentiated the OVA-induced airway inflammation, mucus production and collagen deposition [33,34], mimicking at least in part the responses observed in high-fat diet-induced obesity and genetic models of obesity [13,14,15]. In human nasal epithelial cells, a study demonstrated that MGO significantly increased protein expression of pro-inflammatory cytokines and mucus secretion [35]. Therefore, MGO accumulation has been reported as an aggravating or causative factor in these conditions, but the molecular mechanisms underlying the MGO actions are still a matter of debate. Thus, MGO may be considered a potential enhancer of allergic lung inflammation in the presence of comorbidities such as diabetes and obesity. Interestingly, the classic oral anti-hyperglycemic drug metformin has been reported to scavenge MGO [36,37], which may explain the lower risks of asthma exacerbation in patients taking metformin [38,39].

Empagliflozin, a selective sodium-glucose cotransporter 2 (SGLT2) inhibitor, is widely used in the treatment of type 2 diabetes mellitus [40]. By blocking glucose reabsorption in the renal tubules, it promotes glycosuria hence reducing plasma glucose levels. Beyond glycemic control, clinical studies have demonstrated significant cardiovascular and renoprotective benefits associated with SGLT2 [40,41]. Several pleiotropic effects of SGLT2 inhibitors have been proposed, including their anti-inflammatory and antioxidant properties [42,43]. For instance, in OVA-induced allergic asthma in mice, empagliflozin reduced the airway hyperresponsiveness, cell infiltration, and smooth muscle remodeling [39]. Other SGLT2 inhibitors such as canagliflozin and dapagliflozin share with empagliflozin this anti-inflammatory effect in OVA-induced inflammation, but the mechanism behind this anti-inflammatory effect is not fully understood [44,45]. In a preliminary study carried out in MGO-exposed mice and submitted to OVA-challenge, we observed that empagliflozin treatment mitigated the deleterious effects of MGO on the airways, resulting in marked reduction in cell infiltration. Therefore, we dealt with hypothesis that the protection conferred by SGLT2 inhibitors on allergic airway inflammation may result from a dual mechanism, that is, clearing MGO from circulation and promoting local activation of GLO1. Specifically, in MGO-exposed mice, we aimed to assess whether treatment with the SGLT2 inhibitor empagliflozin reduces the MGO levels in serum and lung tissues, limiting the OVA-induced airway inflammation. To achieve this, mice consuming 0.5% MGO for 12 weeks were treated or not with empagliflozin for 2 weeks, after which animals were intranasally challenged with OVA or instilled with phosphate-buffered saline (PBS). Cell infiltration in bronchoalveolar lavage fluid (BALF) and lung parenchyma, mucus production, collagen deposition, and measurements of OVA-IgE, IL-4, IL-5, IL-13, eotaxin, TNF-α, IL-17, and IL-10 levels were evaluated. The levels of MGO and methylglyoxal-derived hydroimidazolone (MG-H1) and GLO 1 activity in serum and lung tissues were also investigated.

## 2. Results

**Figure 1:**
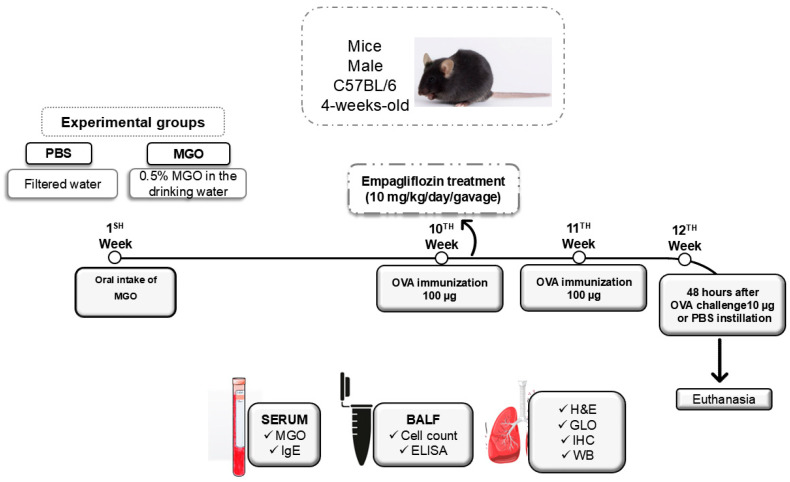
Experimental protocol for immunization and challenge with ovalbumin (OVA): Male C57BL/6 mice were administered oral 0.5% methylglyoxal (MGO) for 12 weeks, with or without subsequent treatment with SGLT2 inhibitors during the final 2 weeks. Certain elements of the figure were created using images from Servier Medical Art, which is licensed under a Creative Commons Attribution 3.0 Unported License (https://creativecommons.org/licenses/by/3.0/ accessed on 10 April 2025). SGLT2, selective sodium-glucose cotransporter 2; BALF, bronchoalveolar lavage fluid; H&C, hematoxylin/eosin; IHC, immunohistochemistry; WB, Western blotting; GLO, glyoxalase.

Figure 1 illustrates the experimental protocols for airway immunization and challenge with OVA in mice under exposed to MGO and treated or not with empagliflozin. Briefly, mice were exposed to 0.5% MGO for twelve weeks and in the last two weeks of this treatment (10th to 12th week), they received concomitantly empagliflozin (10 mg/kg, gavage). On the 10th and 11th week, mice were immunized against OVA and then intranasally challenged with this antigen at the 12th week. At 48 h later, BALF, lung tissues, and circulating blood were collected.

### 2.1. MGO Exposure and Empagliflozin Treatment Do Not Affect Blood Glucose Levels and Body Weight

The fasting glycemia remained within normoglycemic ranges across all groups, regardless of MGO exposure or SGLT2 inhibitor administration, with no significant alterations between groups observed (100 ± 2.1, 101 ± 2.9 and 100 ± 2.3 mg% for untreated-control, MGO and MGO + empagliflozin groups, respectively; *n* = 5). Similarly, the body weight was significantly affected by neither MGO nor empagliflozin treatments (31.7 ± 0.42, 30.9 ± 0.40, and 30.2 ± 0.45 g for untreated-control, MGO and MGO + empagliflozin groups, respectively; *n* = 5).

### 2.2. Analysis of Inflammatory Cell Migration in BALF

**Figure 2:**
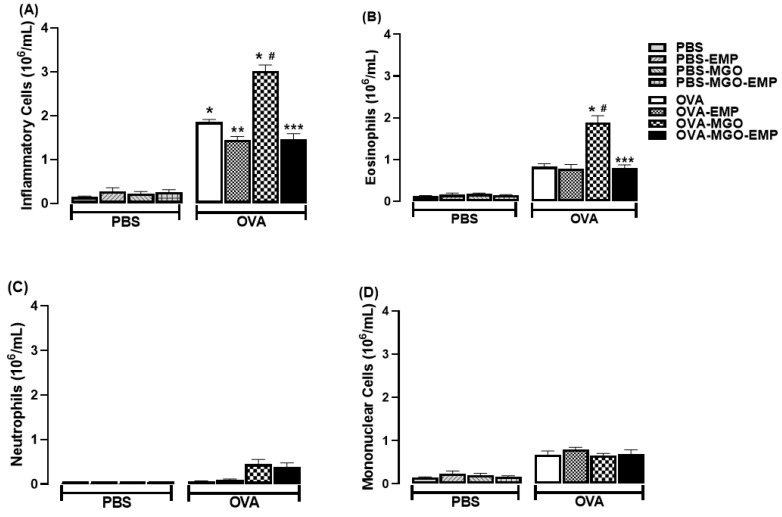
Counts of total inflammatory cells (**A**), eosinophils (**B**), neutrophils (**C**), and mononuclear cells (**D**) in the bronchoalveolar lavage fluid (BALF) of mice intranasally challenged with ovalbumin (OVA). Mice were treated with 0.5% methylglyoxal (MGO) in drinking water for 12 weeks, either alone or in combination with empagliflozin (EMP). The data are expressed as mean ± standard error of the mean (SEM) with n = 5. * *p* < 0.05 compared to respective groups in PBS-instilled mice; ** *p* < 0.05 compared to respective OVA-EMP groups; ^#^ *p* < 0.05 compared to respective OVA groups; *** *p* < 0.05 compared to OVA-MGO groups.

Initially, in PBS-instilled and OVA-challenged mice, the migration of inflammatory cells into the BALF was assessed by quantifying the total number of inflammatory cells, eosinophils, neutrophils, and mononuclear cells across all experimental groups. In animals instilled with PBS, negligible migration of inflammatory cells or eosinophils in BALF was observed with no statistical differences between these groups, regardless of MGO or empagliflozin treatments (Figure 2A–D). As opposed, intranasal challenge with OVA promoted a significant increase in the total number of inflammatory cells and eosinophils counts in BALF, which was markedly potentiated by the MGO exposure (*p* < 0.05, Figure 2A,B). Treatment with empagliflozin alone caused a small but significant reduction on total inflammatory cells (*p* < 0.05), without affecting the eosinophil counts in BALF; however, empagliflozin treatment concomitant with MGO exposure mitigated the migration of total cells and eosinophils, achieving the same levels as OVA-challenge alone (*p* < 0.05, Figure 2A,B). Regarding the counts of neutrophils and mononuclear cells in BALF, no significant differences were detected between groups, regardless of MGO exposure and/or empagliflozin treatment (Figure 2C,D).

### 2.3. Analysis of Inflammatory Cell Migration, Collagen Deposition and Mucus Secretion in the Lung Tissue

**Figure 3:**
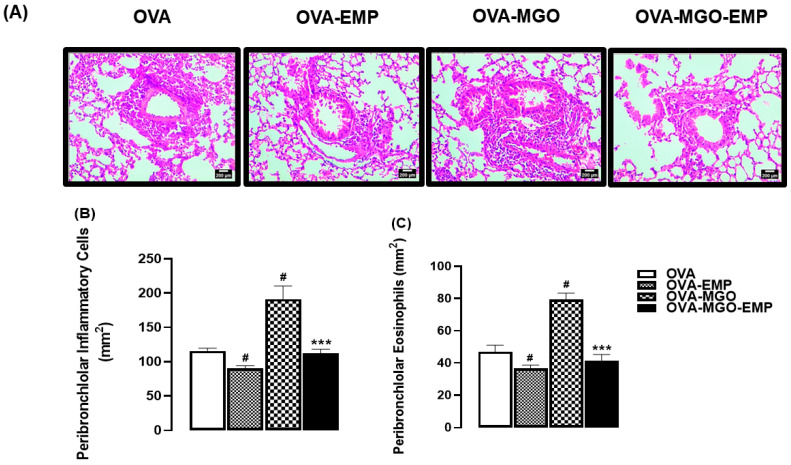
Representative images of hematoxylin and eosin (H&E) staining (**A**), quantification of total inflammatory cells (**B**), and eosinophils (**C**) in lung tissue sections from ovalbumin (OVA)-challenged mice. Mice were treated with 0.5% methylglyoxal (MGO) in drinking water for 12 weeks, either alone or in combination with empagliflozin (EMP). The data are expressed as mean ± standard error of the mean (SEM) with n = 5. ^#^ *p* < 0.05 compared to respective OVA groups; *** *p* < 0.05 compared to the OVA-MGO groups. In panel A, scale bar = 200 μm (200× magnification).

Likewise, in OVA-challenged mice, histopathological assessment using hematoxylin and eosin (H&E) staining revealed a marked accumulation of total inflammatory cells and eosinophils in the peribronchiolar regions, which was significantly increased by the MGO exposure (*p* < 0.05; Figure 3A–C). Treatment with empagliflozin alone caused a small but significant reduction on total inflammatory cells (*p* < 0.05), without affecting the eosinophil counts in the lung parenchyma. However, empagliflozin treatment concomitant with MGO exposure mitigated the migration of total cells and eosinophils in the airways (*p* < 0.05, Figure 3A,B).

**Figure 4:**
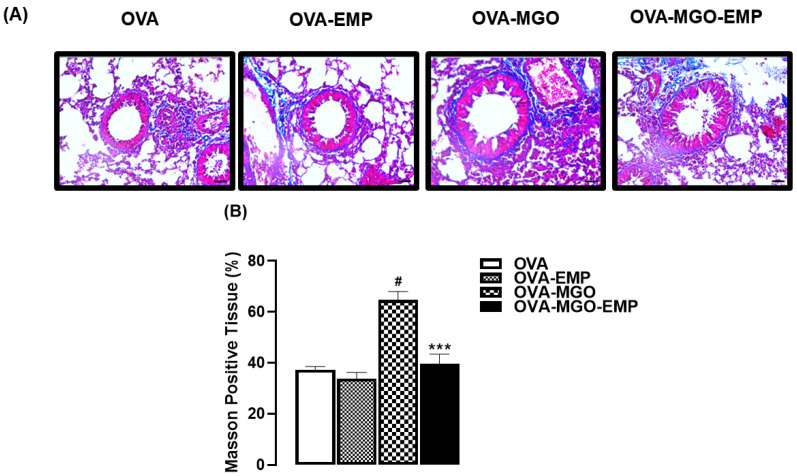
Representative images (**A**) and quantification (%) of collagen deposition (**B**) in lung sections from ovalbumin (OVA)-challenged mice, as assessed by Masson’s trichrome staining. Mice were treated with 0.5% methylglyoxal (MGO) in drinking water for 12 weeks, either alone or in combination with empagliflozin (EMP). The data are expressed as mean ± standard error of the mean (SEM) with n = 5. ^#^ *p* < 0.05 compared to respective OVA groups; *** *p* < 0.05 compared to the MGO-OVA groups.

**Figure 5:**
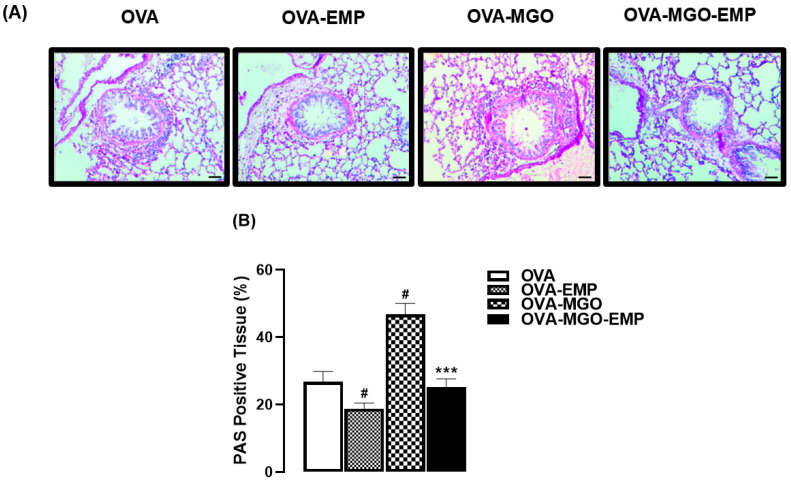
Representative images (**A**) and quantification (%) of mucus production (**B**) in lung sections from ovalbumin (OVA)-challenged mice, as assessed by periodic Acid-Schiff (PAS) staining. Mice were treated with 0.5% methylglyoxal (MGO) in drinking water for 12 weeks, either alone or in combination with empagliflozin (EMP). The data are expressed as mean ± standard error of the mean (SEM) with n = 5. ^#^ *p* < 0.05 compared to respective OVA groups; *** *p* < 0.05 compared to OVA-MGO group.

In OVA-challenged mice, collagen deposition (evaluated by Masson’s trichrome staining; Figure 4A,B) and mucus production (evaluated by periodic acid–Schiff; Figure 5A,B) were significantly increased (*p* < 0.05) by MGO exposure. Treatment with empagliflozin alone caused a small but significant reduction (*p* < 0.05) in mucus production without significantly affecting the collagen deposition; however, empagliflozin treatment concomitant with MGO exposure mitigated the MGO-increases in collagen deposition (Figure 4A,B) and mucus production (Figure 5A,B).

### 2.4. Analysis of IgE, IL-4, IL-5, IL-13 and Eotaxin Levels

**Figure 6:**
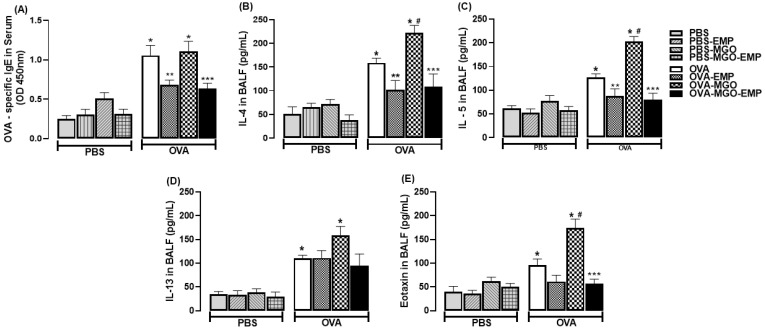
Levels of immunoglobulin IgE in serum (**A**) and IL-4 (**B**), IL-5 (**C**), IL-13 (**D**), and eotaxin (**E**) in bronchoalveolar lavage fluid (BALF) of mice instilled with phosphate-buffered saline (PBS) or intranasally challenged with ovalbumin (OVA). Mice were treated or not with 0.5% methylglyoxal (MGO) in drinking water for 12 weeks, either alone or in combination with empagliflozin (EMP). The data are expressed as mean ± standard error of the mean (SEM) with n = 5. * *p* < 0.05 compared to respective groups in PBS-instilled mice; ** *p* < 0.05 compared to respective OVA-EMP groups; ^#^ *p* < 0.05 compared to respective OVA groups; *** *p* < 0.05 compared to OVA-MGO groups.

The IgE levels in serum were markedly increased in OVA-sensitized mice compared with the non-sensitized group (*p* < 0.05; Figure 6A). MGO exposure did not significantly affect IgE levels in OVA-sensitized mice. Empagliflozin given alone or in combination with MGO exposure significantly reduced the IgE levels (*p* < 0.05; Figure 6A).

The IL-4 levels in BALF were markedly increased in OVA-challenged mice compared with PBS-instilled group (*p* < 0.05; Figure 6B). MGO exposure further increased the IL-4 levels in OVA-sensitized mice (*p* < 0.05). Empagliflozin given alone or in combination with MGO exposure significantly reduced the IL-4 levels (*p* < 0.05; Figure 6B).

The IL-5 levels in BALF were significantly increased in OVA-challenged mice compared with PBS-instilled group (*p* < 0.05; Figure 6C). MGO exposure further increased the IL-5 levels in OVA-sensitized mice (*p* < 0.05). Empagliflozin given alone or in combination with MGO exposure significantly reduced the IL-5 levels (*p* < 0.05; Figure 6C).

The IL-13 levels in BALF were significantly increased in OVA-challenged mice compared with PBS-instilled group (*p* < 0.05; Figure 6D). MGO exposure further increased the IL-13 levels in OVA-sensitized mice (*p* < 0.05). Empagliflozin given alone or in combination with MGO exposure had no effect on IL-13 levels but presents a reduced cytokine level (Figure 6D).

The eotaxin levels in BALF significantly increased in OVA-challenged mice compared with PBS-instilled group (*p* < 0.05; Figure 6E). MGO exposure further increased the eotaxin levels in OVA-sensitized mice (*p* < 0.05). Empagliflozin given in combination with MGO exposure significantly reduced the eotaxin levels (*p* < 0.05; Figure 6E).

### 2.5. Analysis of TNF-α, IL-17 and IL-10 Levels

**Figure 7:**
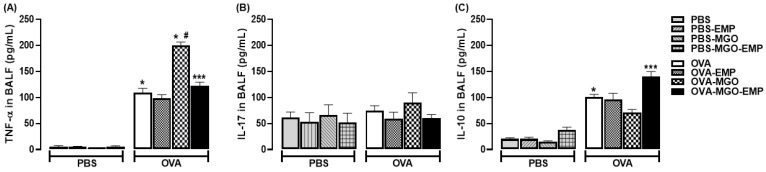
Levels of TNF-α (**A**), IL-17 (**B**) and IL-10 (**C**) in bronchoalveolar lavage fluid (BALF) of mice instilled with phosphate-buffered saline (PBS) or intranasally challenged with ovalbumin (OVA). Mice were treated or not with 0.5% methylglyoxal (MGO) in drinking water for 12 weeks, either alone or in combination with empagliflozin (EMP). The data are expressed as mean ± standard error of the mean (SEM) with n = 5. * *p* < 0.05 compared to respective groups in PBS-instilled mice; ^#^ *p* < 0.05 compared to OVA group; *** *p* < 0.05 compared to OVA-MGO groups.

The TNF-α levels in BALF were markedly increased in OVA-challenged mice compared with PBS-instilled group (*p* < 0.05; Figure 7A). MGO exposure further increased TNF-α levels in OVA-sensitized mice (*p* < 0.05). Empagliflozin given alone had no effect on TNF-α levels but in combination with MGO exposure significantly reduced this cytokine level (*p* < 0.05; Figure 7A).

The IL-17 levels in BALF did not significantly differ between OVA-challenged and PBS-instilled groups (Figure 7B). The levels of IL-17 remained unaffected by MGO exposure alone or in combination with empagliflozin (Figure 7B).

The IL-10 levels in BALF were markedly increased in OVA-challenged mice compared with PBS-instilled group (*p* < 0.05; Figure 7C). MGO exposure did not further increase IL-10 levels in OVA-sensitized mice (*p* < 0.05). Empagliflozin given alone had no effect on IL-10 levels, but in combination with MGO exposure significantly increased this cytokine level (*p* < 0.05; Figure 7C).

### 2.6. SGLT2 Inhibition Reduces MGO and MG-H1 and Increases GLO Activity in Lung or Serum in OVA-Challenged Mice

**Figure 8:**
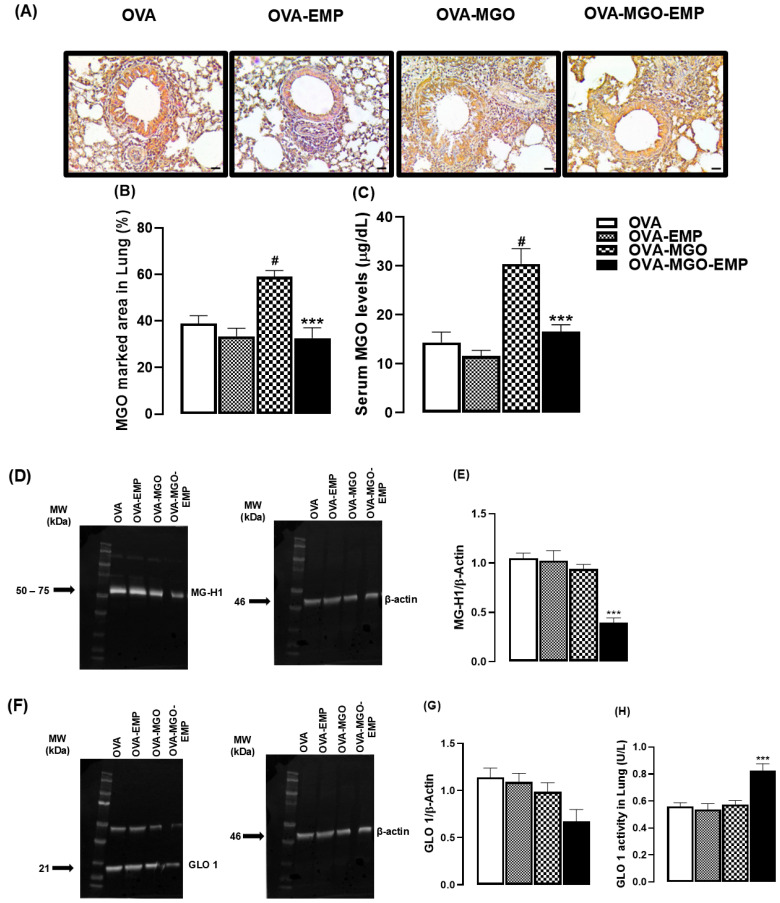
Immunohistochemical analysis of MG-H1 in lung tissue and methylglyoxal (MGO) levels in serum of mice treated with 0.5% MGO in drinking water for 12 weeks, either alone or in combination with empagliflozin (EMP). Representative immunohistochemistry images showing MG-H1 staining in lung tissue are shown in (**A**) with quantification of MG-H1-positive area (%) in lung sections (**B**). Panel (**C**) shows the serum levels of MGO. Panel (**D**) shows the Western blot analysis of MG-H1 expression in lung tissue (**D**) with corresponding densitometric quantification in (**E**). Panel (**F**) shows Western blot analysis of glyoxalase 1 (GLO 1) expression in lung tissue with densitometric quantification in (**G**). Enzymatic activity of GLO 1 in lung tissue is shown in (**H**). The data are expressed as mean ± standard error of the mean (SEM) with n = 5. ^#^ *p* < 0.05 compared to OVA group; *** *p* < 0.05 compared to OVA-MGO groups.

Finally, in OVA-challenged mice, we quantified MGO adducts in lung tissue by both immunohistochemistry (IHC) analysis and by measuring the MGO levels in serum (ELISA assays; Figure 8A–C). The expression and activity of the detoxifying enzyme GLO I in lung tissue were also investigated (Figure 8).

Our results showed a high immunostaining of MG-H1 adduct in lung tissue of MGO-exposed mice compared with control animals (*p* < 0.05). Empagliflozin given alone had no effect on MG-H1 immunostaining, but this treatment suppressed the MGO immunostaining in MGO-exposed mice (*p* < 0.05; Figure 8A,B).

Likewise, the levels of MGO in serum of MGO-exposed mice were significantly higher than those of control animals (*p* < 0.05). Empagliflozin given alone had no significant effect on serum MGO levels but this treatment largely reduced MGO levels in MGO-exposed mice (*p* < 0.05; Figure 8C). When analyzing MG-H1 protein expression in the lung tissue by Western blot, we observed that empagliflozin treatment in MGO-exposed mice markedly reduced the MG-H1 expression in comparison with the other groups (*p* < 0.05; Figure 8D,E).

Furthermore, animals that were challenged with OVA, exposed to MGO, and even treated with empagliflozin did not exhibit either an increase or a decrease in GLO 1 expression in lung tissue (Figure 8F,G). The enzymatic activity of GLO I was significantly increased in lung tissue of animals treated with the empagliflozin in combination with MGO exposure in comparison with the other groups. (*p* < 0.05, Figure 8H).

## 3. Discussion

In this study, using a classical murine model of allergic airway inflammation (AAI) that mimics human asthma, we demonstrated that the SGLT2 inhibitor empagliflozin effectively suppresses OVA-induced airway inflammation in MGO-exposed mice, as evidenced by the marked reductions in eosinophil infiltration, mucus production and collagen deposition, as well as by the reduced levels of IgE, IL-4, IL-5, IL-13, eotaxin, and TNF-α in the lungs. Moreover, empagliflozin efficiently eliminated MGO from circulation and lung tissues, indicating this is the main anti-inflammatory mechanism by which empagliflozin counteracts the harmful effects of MGO exposure on mouse airways.

MGO is a highly reactive dicarbonyl species reported to mediate several types of inflammatory processes [46,47,48]. In the present study, MGO exposure markedly enhanced the recruitment of inflammatory cells, particularly eosinophils, in BALF and peribronchiolar regions of OVA-challenge mice, accompanied by increases in goblet cell metaplasia (mucus production) and collagen deposition, confirming previous studies [33,34]. A study using cultured nasal epithelial cells from asthmatic individuals demonstrated that MGO exposure led to a dose-dependent increase in mucus production [35]. Excessive collagen deposition in the bronchial smooth muscle layer is another hallmark feature of asthma, which is closely associated with airway stiffness leading to aggravated asthma symptoms [49]. Of interest, MGO treatment increases collagen fibril stiffness in mouse tail-tendon fascicle [50].

IgE is a class of immunoglobulin produced by B cells during an immune response, which is switched by the type 2 immunity cytokines IL-4 and IL-13 [5]. High levels of type 2 cytokines (IL-4, IL-5 and IL-13) and eotaxin were detected in BALF of OVA-challenged mice, the levels of which were further increased by MGO exposure. Expectedly, the serum levels of OVA-IgE were markedly increased in OVA-challenged mice, but MGO exposure did not further elevate the IgE levels, suggesting that IgE may have reached its maximal serum levels by OVA-sensitization.

Increased levels of TNF-α have also been detected in the airways of patients with asthma, indicating this pro-inflammatory cytokine plays an important role in the pathogenesis of asthma [51]. In our study, levels of TNF-α elevated significantly in BALF of OVA-challenged mice that was further increased by MGO exposure, which agrees with a previous study showing that MGO-induced glycation of THP-1 macrophages increases the mRNA expression of TNF-α [52].

IL-10 derived from macrophages and other cell types is an anti-inflammatory cytokine that suppresses airway immune responses by inhibiting type 2 cytokines and TNF-α production, which supposedly helps restrain eosinophilic inflammation and bronchial hyperresponsiveness [53]. Despite the macrophage itself being able to generate MGO [54,55,56], IL-10 levels were not significantly affected by MGO exposure, suggesting what MGO may act as a negative regulator of the anti-inflammatory response by suppressing IL-10 production, possibly via inactivation of gene IL-10 receptor, thereby contributing to the amplification of the inflammatory process [56]. Moreover, in obesity-related asthma, Th17 cells via production of pro-inflammatory cytokines and neutrophil chemotactic factors have been implicated in cell recruitment and tissue remodeling [57,58]. In our study, levels of IL-17 in BALF were affected neither by OVA-challenge nor by MGO exposure, which may reflect the predominance of a classical type 2 immunity profile with negligible neutrophil recruitment, accompanied by minimal involvement of type 1 immunity or mixed responses.

Plasma accumulation of MGO in hyperglycemic diabetic patients acts as a potent peptide glycation molecule, giving rise to advanced glycation end products (AGEs) like MG-H1, which is accepted as one of the most important AGE resulting from the nonenzymatic reaction of MGO with arginine residues [20,22]. Accordingly, the serum levels of MGO achieved after a 12-week intake of this compound were markedly higher than vehicle-treated mice. Likewise, immunohistochemical airway sections from OVA-challenged mice exposed to MGO showed an increased tissue immunostaining for MG-H1. This finding confirms that oral MGO intake elevates its own level in circulating blood being distributed to the lung tissue where it glycates arginine residues in proteins forming MG-H1. This is consistent with a previous study in MGO-treated mice for 12 weeks which showed increased plasma MGO levels that positively correlated with free MG-H1 [59]. Interestingly, despite the increased MG-H1 immunoreactivity observed in lung tissue by immunohistochemistry and elevated serum MGO levels, Western blot analysis did not reveal a corresponding increase in MG-H1 modified proteins in lung homogenates by OVA challenged. This discrepancy may be attributed to the nature of MGO as a reactive dicarbonyl metabolite rather than a protein, suggesting that its tissue accumulation does not necessarily result in widespread protein modification detectable by Western blot. Furthermore, immunohistochemistry provides localized detection of MG-H1 accumulation, which may not reflect in total tissue protein lysates.

The SGLT2 cotransporter is selectively expressed in the kidneys [60], with no significant expression detected in other organs. However, several studies have demonstrated beneficial effects of SGLT2 inhibitors on cardiac function and inflammatory processes [40], even in models where SGLT2 is presumed to be absent, suggesting indirect mechanisms of action or involvement of alternative pathways [42]. In our experimental model of OVA-induced asthma combined with MGO exposure, treatment with empagliflozin significantly reduced the recruitment of inflammatory cells and eosinophils in BALF, which was confirmed by the pulmonary morphological analysis. A decrease in both mucus secretion and collagen deposition within the smooth bronchial muscle layer were also observed in empagliflozin-treated mice, indicating an additional protective effect in airway remodeling [44]. The increased levels of IL-4, IL-5, eotaxin and TNF-α in MGO-exposed mice were all normalized by empagliflozin treatment, bringing the responses to the levels of OVA-challenge alone. Another important feature of our study was the ability of empagliflozin to reduce the serum levels of OVA-IgE in OVA-sensitized mice and exposed to MGO. It is also worth mentioning that in animals under no MGO exposure, empagliflozin also produced a discrete (despite significant) anti-inflammatory effect, accompanied by significant reductions in IgE levels in serum. Empagliflozin and canagliflozin also reduced the serum IgE levels in OVA-induced mouse airway disease [61]. The reduction in the in vitro mast cell degranulation by empagliflozin was accepted as the main mechanism responsible for inhibition of both inflammatory cell accumulation and cytokine release in the airways [44,62]. When analyzing the neutrophil recruitment to BALF, empagliflozin treatment modified the cell counts neither in control nor in MGO-exposed mice, suggesting that this SGLT2 inhibitor acts predominantly via the classical type 2 immunity profile with negligible involvement of type 1 immunity or mixed responses, which is consistent with the data of IL-17 levels showing no differences among groups. Additionally, SGLT2 inhibitors promote a shift from the pro-inflammatory macrophage M1 phenotype to the anti-inflammatory M2 phenotype [63]. Accordingly, the empagliflozin treatment elevated significantly the levels of the anti-inflammatory cytokine IL-10 in BALF of MGO-exposed mice, suggesting that increased production of this cytokine may be an efficient mechanism to mitigate the inflammatory process. This finding is in line with previous studies showing that different SGLT2 inhibitors can elevate IL-10 levels in different models of inflammation [64,65,66,67].

Most importantly, however, are our findings showing that empagliflozin treatment can efficiently remove MGO from circulating blood, as demonstrated by reduced levels of both MGO in serum (ELISA assays) and MG-H1 in lung tissue (immunohistochemistry). This is possibly the main mechanism contributing to normalizing the OVA-induced airway inflammation in the MGO-exposed mice. The reduction in MG-H1 levels in lung tissue may be associated with enhanced local detoxification capacity, possibly mediated by increased accumulation or activity of the glyoxalase enzyme, thereby limiting the bioavailability and accumulation of MGO in the inflammatory pulmonary microenvironment. Of interest, empagliflozin treatment (10 mg/kg/day for 6 weeks) was shown to improve dicarbonyl stress parameters, resulting in reduced MGO levels in the kidney cortex of rats [67] suggesting that its protective effects are independent of glycemic control and may result from modulation of dicarbonyl stress. Our findings extend previous evidence to pulmonary inflammation, indicating that SGLT2 inhibitors like empagliflozin may attenuate MGO-driven immunopathology in non-diabetic settings by reducing accumulation of pro-inflammatory metabolic byproducts.

GLO 1 is a key enzyme in the cellular detoxification pathway of MGO that requires glutathione as a precursor and catalyzes the conversion of MGO into D-lactic acid, thereby protecting cells from dicarbonyl stress [68]. In models of lung injury, deficiency or absence of GLO 1 contributes to the progression of the inflammatory process of cystic fibrosis [69]. In MGO-exposed mice, we found that empagliflozin treatment significantly increased enzymatic activity of GLO 1, despite the protein expression remaining unchanged, all suggesting that MGO removal together with enhancement of GLO 1 activity represents an important mechanism by which SGLT2 inhibitors ameliorate inflammatory responses in conditions of elevated levels of dicarbonyl species like MGO. In our study, this finding suggests that empagliflozin may enhance GLO 1 function through post-translational mechanisms rather than by increasing its expression. Moreover, modulates cofactors or signaling pathways involved in the activation of GLO 1, such as the NRF2 or SIRT1/PGC-1α axis, are known to influence inflammation and oxidative stress responses and enzymatic activity [70,71]. In this context, the dual effect of empagliflozin by promoting the elimination of circulating MGO while enhancing GLO 1 activity may prevent the glycation of critical immunoregulatory proteins, such as the IL-10 receptor. The intact IL-10 signaling and production would preserve its anti-inflammatory pathways. Interestingly, in a model of MGO-induced cytotoxicity in adherent neuroblastoma 2a (N2a), the combination of trans-resveratrol and hesperidin (tRES-HESP) significantly elevated the IL-10 levels in serum while restoring the GLO 1 protein levels in N2a cells [72,73], suggesting that elevation of IL-10 levels together with increased GLO 1 activity may represent a protective mechanism mediated by empagliflozin in conditions where the MGO levels are elevated, aiming to maintain the immune homeostasis.

A relevant limitation of this study is the use of the OVA-induced murine model of allergic asthma, which, although widely accepted, does not fully replicate the complexity of human asthma, particularly non-eosinophilic phenotypes and the presence of comorbidities. Although BALB/c mice are traditionally used in type 2 inflammation models as they seem to better mimic the pathophysiology of human asthma, we here used C57BL/6 strain in part because in our hands C57BL/6 mice can mount a robust type 2 immune response [15,33,34], not neglecting that this mouse strain presents natural predisposition toward non–type 2 inflammatory responses, particularly in the context of obesity. Furthermore, currently we are moving toward genetic models of obesity, such as ob/ob mice (leptin-deficient) and db/db mice (leptin receptor–deficient), both of which have a C57BL/6 genetic background. Another limitation of our study refers to GLO 1 enzymatic activity, which was increased following empagliflozin treatment, but we were unable to explore in detail the specific pathways involved in MGO detoxification.

In conclusion, our findings provide novel evidence that empagliflozin efficiently removes MGO from circulation in MGO-exposed mice, while increasing the MGO detoxification by enhancing the GLO 1 enzymatic activity. This is the main mechanism by which empagliflozin suppresses IgE and type 2 immunity cytokines in MGO-exposed mice challenged with OVA, thus emerging as a promising therapeutic strategy, being particularly advantageous in asthma phenotypes linked to metabolic dysregulation, such as obesity and type 2 diabetes mellitus.

## 4. Materials and Methods

### 4.1. Animals

Four-week-old male C57BL/6 mice were obtained from the Multidisciplinary Center for Biological Investigation (CEMIB), University of Campinas (UNICAMP), and housed in groups of three to two per cage in individually ventilated cages (IVCs) under controlled environmental conditions (23 ± 1 °C temperature, 55 ± 5% relative humidity), with a 12 h light/dark cycle. Animals had free access to standard food and water ad libitum throughout the experimental period. All animal procedures and experimental protocols were conducted in accordance with ethical standards and were approved by the Ethics Committee on Animal Use of the University of Campinas (CEUA-UNICAMP; protocol no. 6081-1/2024). The study adhered to the Brazilian Guidelines for the Production, Maintenance, and Use of Animals for Research, as established by the National Council for the Control of Animal Experimentation (CONCEA), in accordance with Normative Resolution No. 30, of 2 February 2016.

### 4.2. Study Design: MGO and SGLT2 Treatments

Animals were treated with 0.5% MGO (Sigma Aldrich, MI, USA) in drinking water ad libitum for a period of 12 weeks, following previously established protocols [33]. Empagliflozin (a selective SGLT2 inhibitor), commercially obtained as Jardiance^®^, was administered by oral gavage at a dose of 10 mg/kg/day during the final two weeks of treatment [67]. On the 12th week, mice were immunized and challenged with OVA to induce experimental asthma (see below).

The study design consisted of eight experimental groups (n = 5 animals per group), organized according to OVA challenge (or PBS instillation) and pharmacological treatments, as follows:

I: PBS (untreated mice intranasally instilled with PBS).

II: PBS-EMP (empagliflozin-treated mice instilled with PBS).

III: PBS-MGO (methylglyoxal-treated mice instilled with PBS).

IV: PBS-MGO-EMP (methylglyoxal- and empagliflozin-treated mice instilled with PBS).

V: OVA (untreated mice instilled with OVA).

VI: OVA-EMP (empagliflozin-treated mice instilled with OVA).

VII: OVA-MGO (methylglyoxal-treated mice instilled with OVA).

VIII: OVA-MGO-EMP (methylglyoxal- and empagliflozin-treated mice instilled with OVA).

### 4.3. Fasting Blood Glucose and Body Weight Analysis

On the 12th week, animals were subjected to metabolic assessments following a 6 h fasting period. Blood samples were collected from the tail vein to evaluate fasting blood glucose levels, which were measured using a handheld glucometer (ACCU-CHEK Performa; Roche Diagnostics, Indianapolis, IN, USA), in accordance with established protocols. In addition, body weight was recorded at the end of the experimental period using a precision digital scale.

### 4.4. Induction of Airway Inflammation via OVA Immunization and Challenge

Mice were actively immunized by subcutaneous injection of 100 μg (OVA, grade V; Sigma-Aldrich, St. Louis, MO, USA) emulsified in 1.6 mg aluminum hydroxide [Al(OH)_3_] in 0.9% sterile saline (total volume 0.4 mL) on days 0 and 7. On days 14 and 15, sensitized animals were intranasally challenged with OVA (10 μg in 50 μL PBS), administered twice daily (at 0 h and again 6 h later), resulting in a total of four OVA challenges over two days. Forty-eight hours after the first challenge, animals were deeply anesthetized and euthanized by isoflurane overdose (>5% concentration) followed by cervical dislocation for confirmation. BALF was then collected, and lung tissues were harvested for further analysis.

### 4.5. Bronchoalveolar Lavage Fluid Collection and Cellular Analysis

Following euthanasia, the trachea was surgically exposed and cannulated using a sterile polyethylene tube attached to a syringe. BALF was performed by instilling 300 μL of sterile PBS into the lungs five consecutive times via the tracheal cannula. The recovered lavage fluids were pooled and centrifuged at 500× *g* for 10 min at 4 °C. The resulting supernatant was collected and stored at −80 °C for subsequent biochemical analysis. The cell pellet was resuspended in 200 μL of PBS and used for total and differential cell counts. Total leukocyte counts were determined using a Neubauer chamber. Differential cell counts were performed on cytospin preparations stained with Diff-Quik^®^, with a minimum of 300 cells classified morphologically as eosinophils, neutrophils, or mononuclear cells.

### 4.6. Quantification of Cytokines, Eotaxin, IgE, and MGO Levels in Serum and BALF

The concentrations of IL-4, IL-5, IL-13, eotaxin, IL-17, TNF-α, and IL-10 in BALF were quantified using commercially available DuoSet ELISA kits (R&D Systems, Minneapolis, MN, USA), according to the manufacturer’s instructions. Serum levels of OVA-specific IgE were determined using a Mouse OVA-Specific IgE ELISA Kit (Catalog No. ANTIA74238-96). Circulating methylglyoxal (MGO) levels were assessed using a competitive ELISA kit (OxiSelect™ Methylglyoxal ELISA Kit, Catalog No. STA-811; Cell Biolabs, San Diego, CA, USA). Optical densities were measured using a Synergy™ H1 Hybrid Multi-Mode Microplate Reader (BioTek Instruments, Winooski, VT, USA).

### 4.7. Histological Processing and Morphometric Analysis of Lung Tissue

Following euthanasia, the lungs were harvested, washed with 10 mL of PBS, and subsequently fixed in 10% phosphate-buffered formalin for 24 h. After fixation, tissues were transferred to 70% ethanol and processed for paraffin embedding. Serial sections (5 μm thick) were obtained using a microtome and stained with H&E for general morphology, Masson’s trichrome for evaluation of subepithelial collagen deposition (fibrosis), and periodic acid–Schiff (PAS) for visualization of mucus production and goblet cell hyperplasia. Histological sections were analyzed by light microscopy using two systems: a Leica DM5000 B microscope (Leica Microsystems, Wetzlar, Germany), equipped with a high-resolution digital camera, and an Optika B-290TB (OPTIKA Srl, Ponteranica, Italy), with a 5.0 MP digital camera for real-time imaging and documentation. Quantitative morphometric analyses were performed independently by two investigators, one of whom was blinded to the experimental groups, to minimize bias. For each staining, five bronchioles per animal were evaluated. Digital images were captured and analyzed using ImageJ 1.54k software (National Institutes of Health, Bethesda, MD, USA; https://imagej.net/ij/docs/install/windows.html, accessed on 10 April 2025). Color-based thresholding was used to measure positive staining areas, which were expressed as area (mm^2^) per bronchiole section.

### 4.8. Immunohistochemistry for MGO Detection in Lung Tissue

For immunohistochemical analysis, paraffin-embedded lung tissue sections were dewaxed in xylene and rehydrated through a graded ethanol series. Antigen retrieval was carried out using 10 mM sodium citrate buffer (pH 6.0) via heat-induced epitope retrieval. Endogenous peroxidase activity was quenched by incubation in 0.3% hydrogen peroxide for 15 min at room temperature. To block nonspecific binding sites, sections were incubated with 5% bovine serum albumin (BSA) for 1 h. Subsequently, the sections were incubated overnight at 4 °C with a monoclonal anti-MGO adduct antibody (1:500 dilution; catalog no. ab243074, Abcam, Cambridge, UK). After rinsing, the sections were incubated with a biotinylated secondary antibody using the Mouse ExtrAvidin-Peroxidase staining kit (1:1000 dilution; catalog no. EXTRA2, Sigma-Aldrich, St. Louis, MO, USA). Immunoreactivity was visualized using 3,3′-diaminobenzidine (DAB) substrate (catalog no. D4293, Sigma-Aldrich), resulting in a brown precipitate indicating positive staining. Images of stained sections were captured using an Optika B-290TB light microscope (OPTIKA S.r.l., Ponteranica, Italy), equipped with a 5.0 MP integrated digital camera, providing real-time imaging and high-resolution documentation.

### 4.9. Protein Extraction and Western Blot Analysis from Mouse Lung Tissue

Lung tissues were homogenized in RIPA lysis buffer (Cat. No. R0278, Sigma-Aldrich, Darmstadt, Germany) supplemented with a protease inhibitor cocktail (10 μL/mL; Cat. No. P8340, Sigma-Aldrich) and incubated for 1 h at 4 °C. Lysates were centrifuged at 12,000× *g* for 15 min at 4 °C, and supernatants were collected for protein quantification using the DC™ Protein Assay Kit I (Cat. No. 5000111EDU, Bio-Rad, Hercules, CA, USA), following the manufacturer’s instructions. Aliquots containing 30 μg of total protein were mixed with 4× Laemmli sample buffer containing 355 mM 2-mercaptoethanol (Cat. No. 161-0747, Bio-Rad) and boiled for 5 min. Proteins were separated by SDS–PAGE and transferred to nitrocellulose membranes using a semi-dry blotting system (Bio-Rad) at 20 V for 20 min. Membranes were blocked overnight at 4 °C in a buffer containing 0.5% non-fat dried milk in TBS-T (10 mM Tris, 100 mM NaCl, 0.02% Tween-20). Membranes were then incubated with the following primary antibodies diluted in 3% BSA: mouse monoclonal anti-MG-H1 (1:1000; Cat. No. STA-011, Cell Biolabs, San Diego, CA, USA), anti-glyoxalase 1 (GLO 1, Cat. No. ab96032, Abcam, Cambridge, UK), and anti-β-actin-peroxidase (1:50,000; Cat. No. A3854, Sigma-Aldrich). MG-H1 and GLO 1 antibodies were incubated overnight at 4 °C, while β-actin was incubated for 1 h at room temperature. After washing, membranes were incubated for 1 h with HRP-conjugated anti-rabbit IgG (1:5000; Cat. No. 7074S, Cell Signaling Technology, Danvers, MA, USA). Bands were visualized using the Clarity™ Western ECL Substrate (Cat. No. 1705061, Bio-Rad), and images were acquired using a Bio-Rad ChemiDoc™ MP Imaging System. Densitometry was performed using Image Lab Software v6.1 (Bio-Rad), and results were expressed as protein-to-β-actin ratios.

### 4.10. Statistical Analysis

Data are presented as mean ± standard error of the mean (SEM). Statistical analyses were performed using GraphPad Prism version 8.0 (GraphPad Software Inc., San Diego, CA, USA). Comparisons among multiple groups were conducted using one-way analysis of variance (ANOVA) followed by Tukey’s post hoc test, while comparisons between two groups were assessed using the unpaired Student’s t-test when applicable. Differences were considered statistically significant at *p* < 0.05.

## 5. Conclusions

In this study, we demonstrated that empagliflozin, an SGLT2 inhibitor, effectively mitigates the allergic airway inflammation in conditions of elevated MGO levels. Given the protection of empagliflozin on type 2 immunity-driven inflammation and airway remodeling, accompanied by an enhancement of the GLO 1 detoxification pathway, SGLT2 inhibition may offer therapeutic potential for asthma, particularly in metabolic contexts of obesity and diabetes. Future research should focus on the clinical applicability of empagliflozin as an adjuvant therapy of asthma associated with metabolic comorbidities.

## Data Availability

The data presented in this study are available on request from the corresponding author due to privacy.

## Abbreviations

The following abbreviations are used in this manuscript:

OVA	Ovalbumin
IgE	immunoglobulin E
MGO	Methylglyoxal
GLO	Glyoxalase
AGE	Advanced Glycation end-Products
SGLT2	Selective Sodium-Glucose Cotransporter 2
PBS	Phosphate-Buffered Saline
BALF	Bronchoalveolar Lavage Fluid
MG-H1	Methylglyoxal-Derived Hydroimidazolone
EMP	Empagliflozin
